# Association Between Intra-Leg Ankle–Brachial Index Asymmetry and Major Adverse Limb Events in Patients with Peripheral Artery Disease

**DOI:** 10.3390/jcm15114263

**Published:** 2026-05-31

**Authors:** Ben Li, Mariam Zakaria, Abdelrahman Zamzam, Shaima AlQrain, Laszlo Göbölös, Nadya Al Matrooshi, Rawand Abdin, Mohammad Qadura

**Affiliations:** 1 Division of Vascular Surgery, St. Michael’s Hospital, Unity Health Toronto, University of Toronto, Toronto, ON M5B 1W8, Canada; benx.li@mail.utoronto.ca (B.L.);; 2 Department of Surgery, University of Toronto, Toronto, ON M5S 1A1, Canada; 3 Temerty Centre for Artificial Intelligence Research and Education in Medicine (T-CAIREM), University of Toronto, Toronto, ON M5S 1A1, Canada; 4 Institute of Medical Science, University of Toronto, Toronto, ON M5S 1A1, Canada; 5 Heart, Vascular, & Thoracic Institute, Cleveland Clinic Abu Dhabi, Abu Dhabi 112412, United Arab Emirates; 6 Department of Medicine, McMaster University, Hamilton, ON L8S 4L8, Canada; 7 Li Ka Shing Knowledge Institute, St. Michael’s Hospital, Unity Health Toronto, University of Toronto, Toronto, ON M5B 1W8, Canada

**Keywords:** ankle–brachial index, asymmetry, intra-leg, prognosis, peripheral artery disease

## Abstract

**Background/Objectives:** The ankle–brachial index (ABI) is the gold-standard test for peripheral artery disease (PAD). However, ABI is conventionally calculated using the higher of the dorsalis pedis (DP)- or posterior tibial (PT)-derived ankle pressure values, which may mask clinically relevant differences between arterial territories within the same limb. We assessed whether intra-leg ABI asymmetry was associated with adverse limb outcomes in patients with PAD. **Methods:** A total of 2495 patients with PAD attending outpatient vascular clinics were retrospectively analyzed. Intra-leg ABI asymmetry was defined as an absolute difference ≥0.15 between DP- and PT-derived ABI values within the same leg. The primary outcome was 2-year major adverse limb events (MALE), defined as major amputation or lower extremity revascularization. Associations between intra-leg ABI asymmetry and outcomes were evaluated using multivariable logistic regression models adjusted for age and sex. **Results:** Overall, 1077 (43%) patients had intra-leg ABI asymmetry. Over 2 years of follow-up, MALE occurred more frequently in patients with intra-leg ABI asymmetry compared to those without (17.2% vs. 7.2%, *p* < 0.001). Patients with intra-leg ABI asymmetry also had higher rates of revascularization (13.9% vs. 5.8%, *p* < 0.001) and major amputation (8.2% vs. 2.3%, *p* < 0.001). In multivariable analysis, intra-leg ABI asymmetry remained independently associated with 2-year MALE (adjusted OR 2.49, 95% CI 1.92–3.23, *p* < 0.001). **Conclusions:** Clinically significant intra-leg ABI asymmetry was common and independently associated with adverse limb outcomes in patients with PAD. These findings suggest that DP–PT ABI discordance may provide important prognostic information beyond conventional ABI reporting methods.

## 1. Introduction

Peripheral artery disease (PAD) affects over 200 million people worldwide and is driven by progressive atherosclerotic stenosis of the lower extremity arteries [1,2]. Although it represents a major cause of mortality and limb loss, PAD remains frequently unrecognized and insufficiently treated in clinical practice [3]. One of the central barriers to improved care is the lack of reliable, standardized methods to facilitate reliable prognostication of high-risk patients to guide vascular evaluation and treatment [3].

The ankle–brachial index (ABI) remains the only widely validated test for PAD [4]. To calculate the ABI for a leg, both the dorsalis pedis (DP) and posterior tibial (PT) artery systolic blood pressures in the ankle are measured [4]. Then, the higher of the DP or PT systolic pressure values is divided by the higher brachial systolic pressure value [4]. In this manner, each leg only has a single reported ABI value that is used to assess for the presence of PAD (ABI < 0.9) and to classify PAD severity (mild [0.7–0.89], moderate [0.4–0.69], and severe [<0.4]) [5]. The limitation of this method is that it does not account for differences between the DP and PT pressure values [5]. For example, a patient with a DP-derived ABI of 1.0 and a PT-derived ABI of 0.7 would be classified as not having PAD because their ABI would be reported as 1.0, which does not meet the PAD criteria of an ABI < 0.9 [5]. However, their PT-derived ABI value of 0.7 suggests potential vascular disease within the PT or more proximal artery, which is masked by the DP-derived ABI of 1.0 [5]. Thus, intra-leg asymmetry in ABI may be an important marker for the presence and progression of vascular disease, which is currently not accounted for in the way that ABIs are reported by vascular laboratories [5]. This is a significant problem because ABIs remain the gold-standard test for PAD [5].

To further understand this problem and to address this clinical gap, we evaluated the clinical significance of discrepancies in intra-leg ABI measurements. Specifically, we assessed whether a clinically relevant discordance of ≥0.15 between the DP- and PT-derived ABI values in the same leg has prognostic value for major adverse limb events (MALE) in patients with PAD [5,6]. Our hypothesis is that patients with intra-leg ABI asymmetry have underlying vascular disease that puts them at increased risk of adverse limb outcomes. If our hypothesis is correct, this would provide important evidence for the need to consider intra-leg ABI asymmetry in the assessment of patients with PAD to guide risk-stratification, further investigations, and treatment decision-making.

## 2. Materials and Methods

### 2.1. Ethical Approval

Ethics approval for this study was granted by the Institutional Review Board of Cleveland Clinic Abu Dhabi on 1 March 2023 (IRB # A-2023-033). Informed consent for participation was not required as per local legislation by the Institutional Review Board of Cleveland Clinic Abu Dhabi given that this was a retrospective analysis of anonymized data. The study procedures adhered to the ethical standards outlined in the Declaration of Helsinki [7].

### 2.2. Study Design

This investigation was conducted as a retrospective cohort study, and results are reported in accordance with the Strengthening the Reporting of Observational Studies in Epidemiology (STROBE) guidelines [8].

### 2.3. Patient Recruitment

Between March 2018 and February 2022, patients with PAD who attended outpatient vascular clinics at Cleveland Clinic Abu Dhabi, United Arab Emirates, were retrospectively analyzed. All clinical assessments, ABI measurements, and follow-up for this study were performed at this institution. PAD was defined as an ABI < 0.9 and absent or diminished pedal pulses in at least 1 leg [9]. Exclusion criteria included acute limb ischemia, acute coronary syndrome, or elevated troponin within the preceding three months. Patients without DP and PT measurements in both legs were excluded.

### 2.4. Baseline Patient Characteristics

Baseline demographic characteristics were obtained for all participants, including age, sex, and body mass index (BMI). ABIs were measured in a certified vascular laboratory by dividing the DP and PT systolic pressure values in each leg by the higher brachial systolic pressure value [4]. For the inclusion of patients with PAD, the higher of the DP- or PT-derived ABI values was used to define an ABI < 0.9, as this is standard clinical practice [4]. Intra-leg ABI asymmetry was defined as an absolute difference between DP- and PT-derived ABI values ≥ 0.15 in the same leg, which has been previously demonstrated to be clinically significant [5,6].

### 2.5. Follow-Up and Outcomes

Outpatient clinic visits were scheduled at 1 year and 2 years post-baseline assessment. The primary outcome was major adverse limb events (MALE) over the 2-year follow-up period. MALE was defined as the necessity for vascular intervention (either open or endovascular lower extremity revascularization) or major lower extremity amputation above the ankle.

### 2.6. Statistical Analysis

Statistical analyses were conducted at both the patient and leg levels. Continuous variables were summarized as mean (standard deviation; SD) and median [interquartile range; IQR], and categorical variables as counts (%). Patient-level baseline characteristics were compared between participants with and without any intra-leg ABI asymmetry using the independent samples t-test for normally distributed variables and the Wilcoxon rank-sum test for non-normally distributed variables. Categorical variables were compared using the chi-square test or Fisher’s exact test when expected cell counts were small (<5). Intra-leg ABI asymmetry was defined using paired DP- and PT-derived ABI measurements within the same leg, with asymmetry classified as present when the absolute difference between DP- and PT-derived ABI values were ≥0.15. For leg-level analyses, left and right legs were combined into a single dataset; comparisons used the same univariate testing framework, and clustered inference was accounted for by fitting generalized estimating equation logistic regression models. Associations between intra-leg ABI asymmetry and clinical outcomes were evaluated using logistic regression models to estimate odds ratios (ORs) with 95% confidence intervals (CIs). Multivariable models were adjusted for age and sex. All tests were two-sided, and statistical significance was set at *p* < 0.05. Python version 3.13.3 was used for all statistical analyses [10].

## 3. Results

### 3.1. Patients

The overall cohort included 2495 patients; 1077 (43%) had intra-leg ABI asymmetry and 1418 (57%) did not. Mean age was 58.7 (SD 13.6) years overall, 57.2 (SD 13.3) years in the no intra-leg asymmetry group, and 60.7 (SD 13.7) years in the intra-leg asymmetry group (*p* < 0.001). There were 728 (29.20%) female patients overall, with 402 (28.39%) in the no intra-leg asymmetry group and 326 (30.27%) in the any intra-leg asymmetry group (*p* = 0.328). Mean BMI was 29.4 (SD 6.2) kg/m^2^ overall, 29.7 (SD 6.1) kg/m^2^ in the no intra-leg asymmetry group and 29.1 (SD 6.4) kg/m^2^ in the any intra-leg asymmetry group (*p* = 0.087). The maximum absolute difference between the DP- and PT-derived ABI values (|DP − PT|) across legs had a median [IQR] of 0.13 [0.07, 0.23] overall, 0.08 [0.05, 0.11] in the no intra-leg asymmetry group and 0.25 [0.19, 0.39] in the any intra-leg asymmetry group (*p* < 0.001) (Table 1).

### 3.2. Events

Overall, 2-year major amputation occurred in 121 (4.85%) patients and was significantly higher in patients in the any intra-leg asymmetry group compared to the no intra-leg asymmetry group (8.17% vs. 2.33%, *p* < 0.001). Similarly, 2-year need for revascularization occurred in 232 (9.30%) patients overall and was significantly higher in the intra-leg asymmetry group compared to the no intra-leg asymmetry group (13.93% vs. 5.78%, *p* < 0.001). Two-year MALE occurred in 287 (11.50%) patients overall, and the rate was significantly higher in the intra-leg asymmetry group compared to the no intra-leg asymmetry group (17.18% vs. 7.19%, *p* < 0.001). These results suggest that adverse limb events occur more frequently in patients with intra-leg ABI asymmetry (Table 2).

### 3.3. Univariable Analysis of the Association Between Intra-Leg ABI Asymmetry and Adverse Limb Events

For 2-year major amputation, intra-leg asymmetry was significantly associated with increased odds (OR 3.734; 95% CI, 2.483–5.618; *p* < 0.001), as was age (OR 1.026; 95% CI, 1.013–1.040; *p* < 0.001). Neither sex (male: OR 1.300; 95% CI, 0.872–1.938; *p* = 0.198) nor BMI (OR 0.999; 95% CI, 0.938–1.065; *p* = 0.986) were statistically significant.

For 2-year need for revascularization, intra-leg asymmetry (OR 2.636; 95% CI, 1.989–3.495; *p* < 0.001) and age (OR 1.037; 95% CI, 1.027–1.047; *p* < 0.001) were significant predictors, whereas male sex (OR 1.053; 95% CI, 0.803–1.382; *p* = 0.706) and BMI (OR 0.989; 95% CI, 0.949–1.030; *p* = 0.596) were not.

For 2-year MALE, intra-leg asymmetry (OR 2.676; 95% CI, 2.071–3.457; *p* < 0.001) and age (OR 1.032; 95% CI, 1.023–1.042; *p* < 0.001) showed significant associations, while male sex (OR 1.101; 95% CI, 0.857–1.415; *p* = 0.452) and BMI (OR 0.993; 95% CI, 0.956–1.032; *p* = 0.722) did not (Table 3).

### 3.4. Multivariable Analysis of the Association Between Intra-Leg ABI Asymmetry and Adverse Limb Events

In multivariable logistic regression models adjusted for age and sex, intra-leg asymmetry remained a strong independent predictor across all outcomes. For 2-year major amputation, intra-leg asymmetry had an adjusted OR of 3.536 (95% CI, 2.343–5.336; *p*-value < 0.001), while age remained significant (adjusted OR 1.018; 95% CI, 1.004–1.032; *p*-value = 0.014) and sex did not (adjusted OR 1.299; 95% CI, 0.848–1.989; *p*-value = 0.229).

For 2-year need for revascularization, intra-leg asymmetry (adjusted OR 2.423; 95% CI, 1.822–3.223; *p*-value < 0.001) and age (adjusted OR 1.028; 95% CI, 1.017–1.039; *p*-value < 0.001) were significant, but male sex was not (adjusted OR 1.166; 95% CI, 0.856–1.589; *p*-value = 0.329).

Similarly, in the model predicting 2-year MALE, intra-leg asymmetry (adjusted OR 2.491; 95% CI, 1.923–3.227; *p*-value < 0.001) and age (adjusted OR 1.024; 95% CI, 1.014–1.034; *p*-value < 0.001) remained significant, while male sex did not (adjusted OR 1.189; 95% CI, 0.896–1.577; *p*-value = 0.231) (Table 4).

## 4. Discussion

### 4.1. Summary of Findings

In this study, we showed that patients with intra-leg ABI asymmetry, defined as an absolute difference between DP- and PT-derived ABI values ≥ 0.15, were more likely to develop 2-year MALE compared to patients without intra-leg ABI asymmetry (17% vs. 7%). These patients are at higher risk for both the need for revascularization (14% vs. 6%) and major amputation (8% vs. 2%). Univariable analysis showed that patients with intra-leg ABI asymmetry were 2.7 times more likely to develop 2-year MALE and 3.7 times more likely to require a major amputation. Multivariable analysis controlling for age and sex showed that this association remained independent and significant, with an adjusted odds ratio of 2.5 for 2-year MALE and 3.5 for major amputation. Thus, intra-leg ABI asymmetry is an important marker for adverse limb events in patients with PAD. Given that over 40% of patients in our study had intra-leg ABI asymmetry, our findings suggest that intra-leg ABI asymmetry should be reported by vascular laboratories and taken into consideration by clinicians for PAD risk-stratification and treatment decision-making. Additionally, further basic, translational, and clinical research is needed to understand the pathophysiology underpinning the association between intra-leg ABI asymmetry and adverse limb events, with the goal of supporting more accurate risk stratification, personalized medical management, and improved outcomes in patients with PAD.

### 4.2. Comparison to Existing Literature

While the prognostic importance of ABI for PAD outcomes has been previously demonstrated [11], no previous work has assessed the association between intra-leg ABI asymmetry and adverse limb events. Secchi and colleagues (2020) used magnetic resonance angiography to assess inter-leg disease symmetry in patients with PAD [12]. While they showed symmetry in disease status between legs, they did not assess intra-leg differences in DP- and PT-derived ABI values, nor their association with PAD outcomes [12]. Pacha and colleagues (2018) showed that patients with an ABI < 0.5 were 2.7 times more likely to require surgical revascularization compared to patients with an ABI > 0.5 [13]. However, they did not assess discordance between DP- and PT-derived ABI values nor its impact on PAD outcomes. Lee and colleagues (2023) showed that Black adults with an abnormal ABI were at approximately two times higher risk of major adverse cardiovascular events and death compared to patients with normal ABIs [14]. McDermott and colleagues showed a significant association between ABI and leg function, with only 40% of patients with an ABI < 0.4 being able to walk continuously for 6 min compared with more than 95% of patients with an ABI between 1.00 and 1.50 [15]. Similarly, Paskiewicz et al. (2021) showed that patients with an ABI ≤ 0.90 versus 1.11 to 1.20 had an approximately fourfold higher risk of chronic limb-threatening ischemia and ischemic leg amputation [16]. Miname et al. (2016) showed that calculating ABI using the lower ankle pressure rather than the higher ankle pressure increased PAD detection rates and was associated with a higher incidence of adverse cardiovascular outcomes [17]. Intra-leg ABI asymmetry may capture a related but distinct vascular phenomenon. Specifically, asymmetry reflects heterogeneity in arterial perfusion between different arterial territories within the same limb, which may indicate focal or segmental disease distribution that is not fully characterized by a single ABI value, regardless of whether the higher or lower ankle pressure is used [18]. For example, two patients may have similar lower ABI values but substantially different degrees of DP–PT discordance, potentially reflecting different patterns of vascular disease and collateralization [18]. Importantly, our study did not directly compare intra-leg ABI asymmetry with alternative ABI calculation strategies, such as calculation using the lower or higher ankle pressure value [17]. Future studies should evaluate the relative and complementary prognostic value of these approaches for predicting PAD outcomes [17]. Our study adds to this evidence by showing that not only are single ABI measurements important in predicting PAD outcomes, but that intra-leg ABI asymmetry also has significant prognostic value for adverse limb events to guide clinical decision-making. These results highlight the importance of further mechanistic research to better elucidate the biological processes underlying the association between intra-leg ABI asymmetry and PAD progression. A more detailed understanding of these pathways may enhance risk stratification and enable the development of targeted therapeutic strategies aimed at preserving limb function and improving outcomes in patients with PAD.

### 4.3. Explanation of Findings

There are several explanations for our findings. Notably, over 40% of patients in our cohort had intra-leg ABI asymmetry. This is likely because the DP and PT are different arteries [18]. The DP comes from the anterior tibial artery, which is a branch of the popliteal artery [18]. In contrast, the PT is a branch of the more distal tibioperoneal trunk, a common site for atherosclerotic disease [18]. Thus, if a patient has disease in their tibioperoneal trunk, they may have a PT systolic blood pressure that is lower than that of the DP [18]. This may explain the high rate of intra-leg ABI asymmetry demonstrated in our study, but further confirmation is needed through dedicated imaging studies, including arterial duplex ultrasound [18]. Our study also showed that patients with intra-leg ABI asymmetry had a higher rate of adverse limb events, potentially because of underrecognized disease [4]. Given that the higher of the DP or PT systolic blood pressure values is used to calculate the ABI, the potentially diseased artery may be masked [4]. For example, if a patient has a DP-derived ABI of 1.0 and a PT-derived ABI of 0.4, they may have severe disease in their PT yet be deemed to have a normal ABI of 1.0, which does not meet the criteria for PAD [4]. As a result, they may not receive appropriate workup, risk-reduction medications, and follow-up [5]. This delay in diagnosis and management may lead to disease progression, the development of chronic limb-threatening ischemia, and eventually the need for surgical revascularization and limb loss [5]. These hypotheses require further confirmation through dedicated basic science and translational studies.

### 4.4. Implications

Our results offer important clinical implications for the assessment and management of patients with PAD. Currently, the ABI is the gold-standard test for PAD [4]. However, the current way that it is reported may mask underlying vascular disease [4]. Traditionally, the ABI has been calculated by taking the higher of the DP or PT systolic blood pressure values divided by the higher of the brachial systolic blood pressure values [4]. As demonstrated in our study, there is often a clinically significant difference between the DP and PT blood pressure values. Thus, taking the higher of the two pressure values may not fully reflect the perfusion of the limb. Historically, the higher of the DP- or PT-derived ABI values was reported because of the hypothesis that extensive collateralization occurs in the foot [19]. Though this may be true for some patients, some individuals may not develop adequate collateralization between their DP and PT due to risk factors such as impaired angiogenesis from smoking, diabetes, or hypertension, among other factors [20,21,22]. Furthermore, the concept of angiosomes states that different parts of the foot are predominantly supplied by different arteries [23]. While the DP predominantly supplies the anterior and dorsal aspects of the foot, the PT predominantly supplies the posterior and plantar aspects of the foot [23]. Thus, disease in one artery but not the other may still lead to inadequate perfusion to one part of the foot, leading to pain, tissue loss, and infection, putting the entire limb at risk for amputation [23]. Thus, the current reporting of the ABI as the higher of the DP- or PT-derived values may not fully reflect the perfusion of the foot.

Our results provide evidence supporting the prognostic importance of intra-leg ABI asymmetry in relation to limb outcomes in patients with PAD. Thus, our findings suggest the importance of reporting both the DP- and PT-derived ABI values in reports generated by vascular laboratories and indicating whether there is a clinically significant intra-leg ABI discrepancy ≥ 0.15. If an intra-leg ABI asymmetry is identified, the patient may benefit from further workup, including arterial duplex ultrasound to assess blood flow and to evaluate disease presence and severity [24]. High-risk patients may benefit from risk reduction medications such as antiplatelets or statins, as well as lifestyle modifications, in addition to close follow-up to regularly assess their limb perfusion and reduce their risk of limb loss [25]. This approach may improve the management of PAD by facilitating more accurate risk stratification and allowing for earlier therapeutic intervention [26]. In turn, it could lead to better patient outcomes while also reducing overall healthcare costs [26]. Further validation of this study’s results is needed to support clinical implementation of the proposed strategy. Of note, this study primarily assessed the prognostic value of intra-leg ABI asymmetry and was not designed to evaluate intra-leg ABI asymmetry as a screening or diagnostic tool in patients without existing PAD, which requires further investigation.

Importantly, intra-leg ABI asymmetry should not be interpreted independently of overall ABI severity [4]. Patients with uniformly reduced DP- and PT-derived ABI values may represent a distinct high-risk phenotype characterized by diffuse or advanced multilevel vascular disease despite minimal asymmetry [27]. Specifically, Pereira Filho et al. (2022) showed that patients with uniformly low ABI values may have a poor long-term cardiovascular prognosis even without asymmetric perfusion measurements [27]. In contrast, marked DP–PT discordance may reflect focal or segmental disease distribution within the limb [4]. Thus, absolute ABI severity and intra-leg ABI asymmetry may provide complementary prognostic information, and future studies should evaluate integrated approaches incorporating multiple ABI-derived parameters for PAD risk stratification to improve predictive accuracy [4]. Specifically, future investigations should evaluate combined classification approaches incorporating both absolute DP- and PT-derived ABI values and DP–PT discordance patterns, including patients with normal ABI values in both arteries, discordant ABI values between arteries, and uniformly reduced ABI values, to determine whether these phenotypes provide incremental prognostic information for PAD outcomes.

### 4.5. Limitations

Although these findings provide useful insights into PAD assessment and risk stratification, several limitations should be considered. First, this study was conducted at a single academic center, which may restrict the generalizability of the results to other populations. Accordingly, future multicenter studies are needed to validate and expand upon these findings. Second, although the cohort of 2495 patients is substantial, larger sample sizes in future research would allow for more robust and generalizable analyses. Third, this study evaluated intra-leg ABI asymmetry at a single baseline timepoint and did not assess longitudinal within-patient changes in asymmetry over time. Progression or worsening of intra-leg ABI asymmetry may provide additional prognostic information beyond a single baseline measurement and should be evaluated in future longitudinal studies. Fourth, residual confounding from unmeasured variables, including diabetes, smoking status, hypertension, dyslipidemia, chronic kidney disease, baseline PAD severity, prior revascularization, and medication use, may have influenced the observed associations, as these factors are associated with PAD outcomes. Future work is underway by our group to incorporate this clinical data into our models to reduce the risk of confounding. Finally, additional imaging and translational investigations are needed to better understand the mechanisms driving intra-leg ABI asymmetry and its relationship with adverse limb outcomes. Moreover, feasibility and cost-effectiveness studies will be important to evaluate the practical implementation of incorporating intra-leg ABI asymmetry into routine clinical reporting and interpretation.

## 5. Conclusions

In this study, we demonstrated that over 40% of patients had intra-leg ABI asymmetry (absolute difference in DP- and PT-derived ABI values ≥ 0.15 in the same leg), which was independently associated with 2-year MALE, including the need for revascularization and major amputation. ABIs are currently reported by taking the higher of the DP- or PT-derived values, which may not fully reflect limb perfusion. Our study provides evidence for the prognostic value of intra-leg ABI asymmetry for adverse limb events, and the importance of assessing for and considering this finding to support clinical decision-making, including further investigations, risk reduction, and treatment. Moreover, these results emphasize the need for continued basic and translational research to better define the biological mechanisms linking intra-leg ABI asymmetry with the onset and progression of PAD. Elucidating these processes may enhance understanding of PAD pathophysiology, support the development of more individualized treatment strategies, and ultimately improve limb outcomes in this vulnerable patient group.

## Figures and Tables

**Table 1:** Baseline characteristics.

	Overall (*n* = 2495)	No Intra-Leg ABI Asymmetry (*n* = 1418)	Any Intra-Leg ABI Asymmetry (*n* = 1077)	*p*-Value
Age (years), mean (SD)	58.7 (13.6)	57.2 (13.3)	60.7 (13.7)	<0.001
Female, *n* (%)	728 (29.20%)	402 (28.39%)	326 (30.27%)	0.328
BMI (kg/m^2^), mean (SD)	29.4 (6.2)	29.7 (6.1)	29.1 (6.4)	0.087
Max |DP − PT| across legs, median [IQR]	0.13 [0.07, 0.23]	0.08 [0.05, 0.11]	0.25 [0.19, 0.39]	<0.001

Abbreviations: ABI (ankle–brachial index), BMI (body mass index), DP (dorsalis pedis), PT (posterior tibial), SD (standard deviation), IQR (interquartile range).

**Table 2:** Events over 2 years of follow-up.

	Overall (*n* = 2495)	No Intra-Leg ABI Asymmetry (*n* = 1418)	Any Intra-Leg ABI Asymmetry (*n* = 1077)	*p*-Value
Major amputation, n (%)	121 (4.85%)	33 (2.33%)	88 (8.17%)	<0.001
Need for revascularization, n (%)	232 (9.30%)	82 (5.78%)	150 (13.93%)	<0.001
MALE composite (major amputation or need for revascularization), n (%)	287 (11.50%)	102 (7.19%)	185 (17.18%)	<0.001

Abbreviations: ABI (ankle–brachial index), MALE (major adverse limb event).

**Table 3:** Univariable analysis of the association between intra-leg ankle–brachial index asymmetry and adverse limb events over 2 years of follow-up.

Outcome	Predictor	OR	95% CI Low	95% CI High	*p* Value
Major amputation	Intra-leg ABI asymmetry	3.734	2.483	5.618	<0.001
	Age	1.026	1.013	1.04	<0.001
	Male sex	1.3	0.872	1.938	0.20
	BMI	0.999	0.938	1.065	0.99
Need for revascularization	Intra-leg ABI asymmetry	2.636	1.989	3.495	<0.001
	Age	1.037	1.027	1.047	<0.001
	Male sex	1.053	0.803	1.382	0.706
	BMI	0.989	0.949	1.03	0.596
MALE	Intra-leg ABI asymmetry	2.676	2.071	3.457	<0.001
	Age	1.032	1.023	1.042	<0.001
	Male sex	1.101	0.857	1.415	0.452
	BMI	0.993	0.956	1.032	0.722

Abbreviations: ABI (ankle–brachial index), MALE (major adverse limb event), BMI (body mass index), OR (odds ratio), CI (confidence interval).

**Table 4:** Multivariable analysis of the association between intra-leg ankle brachial index asymmetry and adverse limb events over 2 years of follow-up.

Outcome	Predictor	Adjusted OR	95% CI Low	95% CI High	*p* Value
Major amputation	Intra-leg asymmetry	3.536	2.343	5.336	<0.001
	Age	1.018	1.004	1.032	0.014
	Male sex	1.299	0.848	1.989	0.229
Need for revascularization	Intra-leg asymmetry	2.423	1.822	3.223	<0.001
	Age	1.028	1.017	1.039	<0.001
	Male sex	1.166	0.856	1.589	0.329
MALE	Intra-leg asymmetry	2.491	1.923	3.227	<0.001
	Age	1.024	1.014	1.034	<0.001
	Male sex	1.189	0.896	1.577	0.230

Abbreviations: ABI (ankle–brachial index), MALE (major adverse limb event), OR (odds ratio), CI (confidence interval).

## Data Availability

The original contributions presented in the study are included in the article; further inquiries can be directed to the corresponding author.
